# Role of Axillary Restaging in Breast Cancer Patients with Preoperative Diagnosis of Isolated Chest Wall Recurrence After Mastectomy: A Literature Review

**DOI:** 10.3390/medicina62020273

**Published:** 2026-01-28

**Authors:** Niña Xiamina Alger-Turrecha, Tessa Ying Zhen Tan, Geok Hoon Lim

**Affiliations:** 1 Breast Department, KK Women’s and Children’s Hospital, Singapore 229899, Singapore; nina.alger.turrecha@kkh.com.sg (N.X.A.-T.);; 2 Duke-NUS Medical School, 8 College Road, Singapore 169857, Singapore

**Keywords:** breast cancer, chest wall recurrence, sentinel lymph node biopsy, mastectomy, axillary lymph node dissection

## Abstract

*Background and Objectives:* Chest wall recurrence can occur infrequently after mastectomy in breast cancer patients. While wide excision of chest wall recurrence is indicated in operable patients without metastasis elsewhere, management of the axilla remains controversial. We reviewed the literature to determine the role of axillary staging in patients with a preoperative diagnosis of isolated chest wall recurrence. *Materials and Methods:* A PubMed search was performed for relevant articles dated between 1 January 2000 and 31 December 2024. Only English articles comprising female patients with invasive chest wall recurrence after mastectomy were included. Patients with concomitant metastasis elsewhere, no operation for recurrence and no oncological outcomes were excluded. The outcomes were compared between patients with or without axillary staging during recurrence. *Results*: A total of 15 studies with 485 eligible patients were analyzed. Of these patients, 242 (49.9%), 182 (37.5%), 53 (10.9%), and 8 (1.6%) patients had sentinel lymph node biopsy (SLNB), no axillary surgery, failed SLNB, and upfront axillary lymph node dissection (ALND), respectively, for restaging. Among operated patients with reported axillary status, 33/231 (14.3%) had metastatic nodes on axillary staging. On follow-up of 38.2 months (range: 10–61.2), 101/485 (20.8%) patients developed a second recurrence, of which 16/447 (3.6%) developed ipsilateral axillary recurrence. Ipsilateral axillary recurrence among patients with and without axillary surgery was 12/182 (6.6%) and 4/265 (1.5%), respectively. *Conclusions:* Ipsilateral axillary recurrence was low in patients with or without axillary restaging on medium-term follow-up. Due to the heterogeneity of the studies, larger studies with longer follow-up periods are needed to determine the best management for the axilla.

## 1. Introduction

Breast cancer is the most common cancer among women worldwide. While the prognosis for breast cancer has improved with the use of multiple treatment modalities, such as surgery, chemotherapy, radiotherapy, endocrine therapy, human epidermal growth factor receptor 2 (HER2)-targeted therapy, immunotherapy, etc., there remains a risk of recurrence.

A recurrence can occur locally, regionally or distantly with a higher risk of recurrence in patients with unfavorable subtypes such as triple negative or HER2-positive [1,2]. Isolated local recurrence has been reported to occur in about 5–10% and 1–3% of patients after breast-conserving surgery and mastectomy, respectively [3,4].

After mastectomy, 90% of chest wall recurrences occur within the first 5 years of surveillance, though later recurrences can be seen in patients with hormone-positive subtypes on endocrine treatment [5]. In patients with recurrent breast cancer, genetic testing should be offered to exclude a genetic cause for the recurrence [6]. Factors that could increase the risk of chest wall recurrences include advanced stage, younger age, and lymphovascular invasion [7]. Radiotherapy could be given in high-risk patients after mastectomy to reduce the risk of recurrences [8].

Of these chest wall recurrences, 40–70% are isolated, but may also be associated with concomitant regional recurrences in 30–40% of cases or have distant metastasis at the time of diagnosis or within a few months of diagnosis [5].

Isolated local recurrence has also been reported to have a better outcome compared to patients with regional or distant metastasis [9,10], so isolated local recurrence should be treated with curative intent with complete resection.

In patients with a preoperative diagnosis of ipsilateral breast tumor recurrence following breast-conserving surgery, the management of the axilla is not well established, though an axillary restaging, usually with sentinel lymph node biopsy (SLNB), may be considered at the time of resection of the ipsilateral breast tumor recurrence [11] in cases of prior SLNB. This axillary restaging could allow assessment of the lymph node status and may alter the patient’s management strategy and prognosis if the SLNB were positive [12].

In patients with a preoperative diagnosis of isolated chest wall recurrence after mastectomy, without clinical or radiological suspicion of metastasis elsewhere, the use of axillary restaging remains controversial. Historically, an axillary lymph node dissection (ALND) was typically performed for axillary restaging during the resection of the chest wall recurrence if an ALND was not done previously [13]. However, ALND can be associated with multiple morbidities such as arm lymphedema, etc. [14].

As a result, in patients with no preoperative clinical evidence of axilla involvement, there was a paradigm shift from ALND to SLNB, reserving ALND for patients with nodal recurrence [15].

In patients with chest wall recurrence, though restaging with SLNB is technically challenging but possible [16,17], there is sparse data on its benefits. The National Comprehensive Cancer Network (NCCN) guidelines on the axillary management of breast cancer patients with isolated chest wall recurrence are not definitive too [11].

We aimed to perform a narrative review of oncological outcomes in patients with a preoperative diagnosis of operable isolated invasive chest wall recurrence with or without axillary restaging. This is the first-reported study, to the best of our knowledge. We present the following article in accordance with the narrative review reporting checklist.

## 2. Materials and Methods

A search on PubMed was performed for relevant articles dated between 1 January 2000 and 31 December 2024, using the following search terms: breast cancer, mastectomy, chest wall recurrence, survival, recurrence, and axillary staging (Table 1). Only articles in the English language comprising female breast cancer patients were included. Articles with no abstract, such as editorials and letters to the editor were excluded.

This review included patients with a preoperative diagnosis of isolated invasive chest wall recurrence after mastectomy. The oncological outcomes of these patients after the operation for recurrence were compared between patients who had axillary restaging surgery versus those without at the time of recurrence. Patients with ductal carcinoma in situ (DCIS) recurrence, concomitant regional or distant metastasis at time of recurrence, no operation for recurrence, and recurrence after lumpectomy were excluded. In addition, patients with no follow-up oncological data after the operation for recurrence, such as local, nodal or distant recurrences, or survival were excluded. Publications with duplication of the study subjects were excluded too.

Two authors conducted the PubMed search independently by first assessing the abstract for relevance. If found to be relevant, the full article was assessed for retrieval of relevant data. For the relevant articles, their references were also examined for relevance. For any relevant publication, a search for similar related articles was also conducted on PubMed. In the event of discordance, the article was reassessed together with another author to reach a consensus.

## 3. Results

After initial assessment of the abstracts for relevance, 55 articles were found. Of these studies, 25 studies had to be excluded as there were no oncological outcomes reported specifically for patients with chest wall recurrence. Another 14 studies were excluded as they only included patients with prior breast-conserving surgery. Of the remaining 16 studies, 1 study [4] had duplication of patients, hence it was excluded.

A total of 15 studies were included for analysis (Table 2). Of these studies, only 6 studies [18,19,20,21,22,23] solely included patients with prior mastectomy. Of these six studies, 379 patients were eligible for analysis. The remaining nine studies [24,25,26,27,28,29,30,31,32] consisted of patients who underwent prior mastectomy or breast-conserving surgery, of which only those with reported oncological outcomes of the former group, with 106 eligible patients, were analyzed. Of these nine studies, one study [25] had duplication of patients with another two studies [26,27]. No patients from this study [25] were analyzed to avoid duplication of patients. However, it was included because it provided the survival outcomes of the patients with prior mastectomy which were not reported in the other two studies. All were retrospective studies, with one case report [19] with a total of 485 eligible patients.

All studies, except one [18], were based on the use of SLNB in patients with recurrence. This one study [18] focused on the outcome for no axillary staging in this group of patients and this contributed to most of the patients with no axillary staging in this review.

After the diagnosis of local chest wall recurrence, axillary imaging was performed with axillary ultrasound in eight studies while it was not mentioned in the other seven studies.

During chest wall recurrence, 242 (49.9%), 53 (10.9%), and 8 (1.6%) patients had SLNB, failed SLNB, and upfront ALND, respectively, on restaging, while 182 (37.5%) patients had none. Of the 242 patients with SLNB, 20 (8.3%) had concurrent ALND, of which 18 were performed for metastatic disease on SLNB. The other two patients had ALND despite non-metastatic SLNB, based on surgeons’ discretion. Of the 231 patients who had axillary surgery and reported axillary nodal status, 33 (14.3%) had metastatic axillary nodes on axillary staging.

Of the 53 patients with failed SLNB, 8 (15.1%) proceeded to ALND, whereas 19 (35.8%) had no further axillary operation, while for the remaining 26 (49.1%) patients, there was no mention of what was performed after the failed SLNB.

On follow-up of 38.2 months (range: 10–61.2), 101/485 (20.8%) patients developed a second recurrence. Of these patients, only 447 patients had data on ipsilateral axillary recurrence. A total of 16/447 (3.6%) patients developed ipsilateral axillary recurrence. Of these 16 patients, 12 did not have axillary surgery, while 4 had axillary staging, of which 3 had a negative nodal status, while one patient’s nodal status was not reported. As a result, the rate of ipsilateral axillary recurrence among patients with no axillary surgery and axillary surgery was 12/182 (6.6%) and 4/265 (1.5%), respectively.

Only three studies reported survival outcomes, with a re-recurrence-free survival rate of 90.2% at 2 years and 81.3% at 4 years [21]. Vicini et al. [20] reported an overall survival rate of 96.7% and a disease-free survival rate of 84.4% at 44.4 months follow-up while a 5-year distant-recurrence-free survival rate of 83% was reported by Poodt et al. [25]. All of these studies included only patients who had axillary staging.

## 4. Discussion

A total of 15 studies with 485 patients were included in this review. In breast cancer patients who developed chest wall recurrence after mastectomy, 242 (49.9%), 182 (37.5%), 53 (10.9%), and 8 (1.6%) patients had SLNB, no axillary surgery, failed SLNB, and upfront ALND, respectively, for restaging. Of the 231 patients who had axillary surgery and a reported axillary nodal status, 33 (14.3%) had metastatic axillary nodes on axillary staging. Only 447 patients had data on ipsilateral axillary recurrence. The rate of ipsilateral axillary recurrence among patients with no axillary surgery and axillary surgery was 12/182 (6.6%) and 4/265 (1.5%), respectively. This is the first reported review comparing the oncological outcomes of chest wall recurrence with or without axillary staging, to the best of our knowledge.

The prevalence of isolated chest wall recurrence after mastectomy is rare [33], hence there is sparse data on its management. As a result, the management of the axilla in patients with a preoperative diagnosis of isolated chest wall recurrence is unclear. In contrast, there is more published data on the management of the axilla in patients with ipsilateral breast recurrence following breast-conserving surgery. While axillary staging may be considered in patients with ipsilateral breast recurrence after breast-conserving surgery [34], it remained uncertain if this axillary approach can be extrapolated to patients with chest wall recurrence. This was because breast surgery for primary cancer has effects on axillary lymphatic drainage and could affect the feasibility of subsequent axillary staging.

Different from breast-conserving surgery, mastectomy is usually indicated for patients with larger tumor sizes [35]. As a result, there is also a higher incidence of nodal metastasis and ALND [36,37]. This often leads to scarring in the axilla and coupled with the lack of breast tissue after mastectomy, a higher rate of aberrant lymphatic drainage was observed compared to patients with prior breast-conserving surgery. In fact, an aberrant rate as high as 77% had been reported in patients with prior mastectomy [38]. The aberrant sites of drainage included the contralateral axilla or/and internal mammary chains, etc. [39]. This hence raises the question if an ipsilateral axilla SLNB was necessary.

Despite a high reported rate of aberrant lymph drainage after mastectomy, a staging SLNB could still be performed during the recurrence setting with a success rate of 63.6–80% [16,22] and 76.9% [16] in patients with prior mastectomy and SLNB or ALND, respectively. In fact, a success rate as high as 93.4%, for axillary SLNB restaging in patients with chest wall recurrence and no prior ALND, has been reported in a specialized center [20].

Though SLNB restaging for chest wall recurrence could be performed despite its technical challenges, its accuracy was not widely reported, since most of this data were based predominantly on patients with prior breast conservation. Nonetheless, it was reported to be accurate with a low false negative rate of 0.2% [38], despite a lower SLNB identification rate and higher aberrant lymphatic drainage in the recurrent setting. In patients with non-metastatic SLNB during the local recurrence setting, the ipsilateral axillary recurrence rate was also low at 1.0% [27], after a median follow-up of 4.7 years. These promising results, however, need to be validated in future studies involving more patients with chest wall recurrence after mastectomy.

Besides sparse data on the accuracy of SLNB, it remained controversial if SLNB would be beneficial for all patients with chest wall recurrence and clinically negative axilla. While some would argue that SLNB was needed as it could change adjuvant treatment decisions, there remained limited data in this area for patients with chest wall recurrence. In the current era when the treatment decision is more dependent on the molecular subtype and/or genomic tests [40,41], this role of SLNB for the decision on the receipt of chemotherapy and hormonal therapy may not be necessary. In addition, radiotherapy would generally be offered for patients with chest wall recurrence unless the patient has had radiotherapy previously [42].

There is also little data regarding the oncological safety for and against SLNB staging in patients with chest wall recurrence, though axillary staging could potentially improve locoregional control and decrease recurrence. However, this needs to be balanced with the potential morbidity and technical challenges associated with axillary restaging. In this review, the rate of ipsilateral axillary recurrence among patients with no axillary surgery and axillary surgery was low in both groups. This finding was similar to another study which consisted of predominantly patients with prior breast-conserving surgery and local recurrence [13]. In patients with failed SLNB on restaging, there was also a low risk of subsequent regional recurrence [26]. All these implied that restaging axillary surgery may not yield additional oncological benefits. However, the latter study comprised mostly patients with prior breast-conserving surgery and local recurrence, hence these findings will need to be validated in a larger cohort of patients with recurrence after mastectomy. However, to date, there is no survival data comparing axillary restaging versus no restaging for patients with prior mastectomy and chest wall recurrence.

To better stage the axilla in the recurrent setting, imaging modalities such as axillary ultrasound have been used preoperatively to detect axillary metastasis. It has been reported to have a sensitivity and specificity of 92.7% and 93.9%, respectively [43]. This could allow those patients with axillary metastasis to be identified preoperatively so that they could have an ALND. Conversely, in patients with a negative axilla ultrasound, SLNB could potentially be omitted. However, in our review, axillary ultrasound was not used universally.

Incidentally, axillary nodal metastasis may also be picked up on the metastatic workup, which is required for patients with recurrent breast cancer [44]. This could include (fluorodeoxyglucose positron emission tomography/computed tomography) FDG-PET/CT [45], which has demonstrated a low risk of metastatic SLNB on restaging if the FDG-PET/CT was negative. This could then allow for a tailored approach of an omission of SLNB restaging in this group of patients, hence reducing morbidity to the axilla.

Strengths of this paper included this being the first reported review comparing the oncological outcomes of chest wall recurrences with or without axillary staging. As there is currently limited data in this area, this review of the relevant studies could provide an insight based on a larger patient cohort.

However, this paper is not without limitations. All of the included studies were retrospective in nature hence there may be selection bias in the treatment which could affect oncological outcomes. Most of the studies also included patients with local recurrence after breast-conserving surgery. As a result of the heterogeneity of the patient cohort, data for patients with chest wall recurrences after mastectomy in these studies may not be specifically detailed, hence excluding some of these patients without oncological data from analysis in this review. There was also a lack of data in the included studies on patients’ tumor profile and morbidity caused by axillary staging, hence these parameters were not reported in this review. In this review, the patients with failed SLNB were analyzed together with the patients who had SLNB or upfront ALND, though some of the patients with failed SLNB did not have further axillary surgery. This was because there were too few patients in the failed-SLNB group to be analyzed as a group. Only one paper in this review contributed to the majority of patients with no axillary surgery while the rest of the papers reported on the outcomes of restaging SLNB. This skewed contribution made further statistical analysis, needed for meta-analysis or systematic review, difficult. Survival outcomes were also not widely reported in the included studies. Finally, only English language articles from PubMed were included in this review, hence relevant articles in other databases or languages may not have been captured.

## 5. Conclusions

In conclusion, there is sparse data in the literature on the management of the axilla in patients with chest wall recurrence and no preoperative evidence of regional or/and distant metastasis. In patients who had axillary staging and a reported nodal status, 14.3% had metastatic axillary nodes. The rate of ipsilateral axillary recurrence, with or without axillary restaging, was low on medium-term follow-up. Due to the heterogeneity of the studies, future larger studies with longer follow-up terms are needed to determine the best management for the axilla.

## Figures and Tables

**Table 1:** The search strategy summary.

Items	Specification
Date of search	21 August 2025
Databases and other sources searched	PubMed
Search terms used	Breast cancer, chest wall recurrence, local recurrence, recurrent breast cancer, mastectomy, axillary surgery, sentinel lymph node biopsy, axillary clearance, lymph node dissection, survival, recurrence
Timeframe	1 January 2000–31 December 2024
Inclusion and exclusion criteria	Included: Studies with female patients who had (I) preoperative diagnosis of invasive chest wall recurrence after mastectomy, without suspicion of metastasis elsewhere, and underwent (II) SLNB/no axillary surgery/failed SLNB/upfront ALND with reported (III) oncological outcomes Excluded: (I) Publications that were not in English, (II) publications with duplication of the study subjects (III), publications that did not have abstracts or patients’ data, etc.
Selection process	Two authors independently performed the search. In cases of discordance, the article was reassessed with another author to reach a consensus.
Any additional considerations if applicable	When a publication has met the inclusion criteria, its references were also reviewed for relevance. Similar related articles were also conducted for all relevant articles.

**Table 2:** Included studies in chronological order based on year of publication. NA—not applicable. NR—not reported. ALND—axillary lymph node dissection. SLNB—sentinel lymph node biopsy. IMN—internal mammary node.

Author/ Year	Number of Patients with Restaging Axillary Surgery	Number of Patients with Metastatic LN on Restaging	Axillary Imaging	Median Months of Follow-Up	Ipsilateral Axillary Recurrence	Other Recurrences	Survival	Other Comments
Lim et al. [18]/2024	No: 181	NA	Ultrasound in 17.5%	59.5	11/181 in no-axilla-surgery group (6.1%) None in axillary surgery group	37.6% in no-axilla-surgery group 7.7% in axillary surgery group	NR	194 mastectomy patients for analysis
	SLNB: 5	0						
	Failed SLNB: NR	NA						
	ALND: 8	1						
Torras et al. [24]/2024	No: 0	NA	Ultrasound or MRI	19.5	1	1 local recurrence	NR	6 mastectomy patients for analysis
	SLNB: 6	NR						
	Failed SLNB: 0	NA						
	Upfront ALND: 0	NA						
Schulze et al. [21]/2023	No: 0	NA	NR	28	0	8 local/distant recurrences, 6/65 in SLNB group, 2/8 in failed-SLNB group	re-recurrence-free survival was 90.2% at 2 years and 81.3% at 4 years	73 mastectomy patients for analysis
	SLNB: 65	10 (5 had ALND)						
	Failed SLNB with no further surgery: 7	NA						
	Failed SLNB so ALND/axillary sampling: 1	NR						
	Upfront ALND:0	NA						
Vicini et al. [20]/2022	No: 0	NA	Ultrasound 100%	44.4	1/82—had negative SLNB	4/82—local regional recurrence 3/82—local regional recurrence and distant metastasis 7/82—distant metastasis	overall survival—96.7% disease-free survival—84.4% for 82 patients	83 mastectomy patients. No outcome for 1 patient with failed SLNB, hence 82 patients for analysis
	SLNB: 82	12 (11 had ALND)						
	Failed SLNB: 1	NR						
	Upfront ALND: 0	NA						
Poodt et al. [25]/2019	No: 0	NA	Ultrasound	61.2 for breast-conserving surgery and mastectomy patients	NR	NR	5-year distant-recurrence-free survival of 83%	61 mastectomy patients—38 of the SLNB group and 12 of the failed-SLNB group were duplicate patients. No outcome for the remaining 11 patients, hence no patients for analysis
	SLNB: 49	7						
	Failed SLNB: 12	NR						
	Upfront ALND: 0	NA						
Poodt et al. [26]/2019	No: 0	NA	Ultrasound	61.2 for breast-conserving surgery and mastectomy patients	0	0 regional recurrence	NR	12 mastectomy patients with unsuccessful SLNB for analysis
	SLNB: 0 Failed SLNB with no further surgery: 9	NA						
	Failed SLNB so ALND/axillary sampling: 3	NR						
	Upfront ALND: 0	NA						
Poodt et al. [27]/2018	No: 0	NA	Ultrasound	56.4 for breast-conserving surgery and mastectomy patients	NR	2/38 regional recurrence	NR	38 mastectomy patients with successful negative SLNB for analysis
	SLNB: 38	0						
	Failed SLNB: 0	NA						
	Upfront ALND: 0	NA						
Johnson et al. [22]/2016	No: 0	NA	NR	55.5	1 patient with failed SLNB and axilla sampling which was negative	No local or supraclavicular recurrence. 1 IMN recurrence in 1/9 SLNB-negative patients	NR	12 mastectomy patients for analysis; 10 had no 2nd recurrences
	SLNB: 10	1						
	Failed SLNB with no further surgery: 1	NA						
	Failed SLNB so ALND/axillary sampling: 1	0						
	Upfront ALND:0	NA						
Karanlik et al. [28]/2016	No: 0	NA	NR	Mean of 36 for breast-conserving surgery and mastectomy patients	0	No local recurrence	NR	31 mastectomy patients for analysis
	SLNB: 10	5						
	Failed SLNB: 21	NR						
	Upfront ALND: 0	NA						
Uth et al. [29]/2015	No: 0	NA	Ultrasound 100%	Mean of 38 for breast-conserving surgery and mastectomy patients	0	NR	NR	14 mastectomy patients for analysis
	SLNB: 9	NR						
	Failed SLNB: 5	NR						
	Upfront ALND: 0	NA						
Vicente et al. [19]/2010	No: 0	NA	NR	33	0	0	NR	1 mastectomy patient for analysis
	SLNB: 1	1—also had ALND						
	Failed SLNB: 0	NA						
	Upfront ALND: 0	NA						
Derkx et al. [30]/2010	No: 11	NA	Ultrasound in 14/70 patients with breast-conserving surgery and mastectomy patients	24 for breast-conserving surgery and mastectomy patients	1 in the no-axilla-surgery group, no recurrence in the 1 patient with SLNB to ALND (20 months follow-up)	NR	NR	18 mastectomy patients. No outcome details for the 6 patients with upfront ALND or remaining 10 patients with no axillary staging, hence 2 patients for analysis
	SLNB: 1	1—also had ALND						
	Failed SLNB with no further surgery: 0	NA						
	Failed SLNB so ALND/axillary sampling: 0	NA						
	upfront ALND: 6	NR						
Karam et al. [23]/2008	No: 0	NA	NR	Mean of 33.3 for 20 mastectomy patients	1/10 patient with negative SLNB had concurrent axillary and local recurrence	1/10 patient with negative SLNB had distant metastasis	NR	Only 17 mastectomy patients with recurrence analyzed. All 5 patients with failed SLNB and 2 patients with SLNB and ALND—no recurrence
	SLNB: 12	2						
	Failed SLNB with no further surgery: 2	NA						
	Failed SLNB so ALND/axillary sampling: 3	NR						
	Upfront ALND: 0	NA						
Boughey et al. [31]/2006	No: 0	NA	NR	13 for breast-conserving surgery and mastectomy patients	0	NR	NR	4 mastectomy patients. No outcome for 2 patients with failed SLNB, hence 2 patients analyzed
	SLNB: 2	0, but had ALND						
	Failed SLNB: 2	NR						
	Upfront ALND: 0	NA						
Roumen et al. [32]/2006	No: 0	NA	NR	10	0	0	NR	1 mastectomy patient analyzed. She had exploration of contralateral axilla
	SLNB: 1	0						
	Failed SLNB with no further surgery: 0	NA						
	Failed SLNB so ALND/axillary sampling: 0	NA						
	Upfront ALND: 0	NA						

## Data Availability

The data sets used and/or analyzed during the current study are available from the corresponding author on reasonable request.

## Abbreviations

The following abbreviations are used in the manuscript:

SLNB	Sentinel lymph node biopsy
ALND	Axillary lymph node dissection
HER2	Human epidermal growth factor receptor 2
NCCN	National Comprehensive Cancer Network
DCIS	Ductal carcinoma in situ
NA	Not applicable
NR	Not reported
IMN	Internal mammary node
FDG PET/CT	Fluorodeoxyglucose positron emission tomography/computed tomography
